# Non-Destructive Methodology to Determine Modulus of Elasticity in Static Bending of *Quercus mongolica* Using Near-Infrared Spectroscopy

**DOI:** 10.3390/s18061963

**Published:** 2018-06-18

**Authors:** Hao Liang, Meng Zhang, Chao Gao, Yandong Zhao

**Affiliations:** 1 School of Technology, Beijing Forestry University, Beijing 100083, China; lianghao@bjfu.edu.cn (H.L.); zhangmeng1996@bjfu.edu.cn (M.Z.); gaochao9158@sina.com (C.G.); 2 Beijing Laboratory of Urban and Rural Ecological Environment, Beijing 100083, China; 3 Key Lab of State Forestry Administration for Forestry Equipment and Automation, Beijing 10083, China

**Keywords:** near-infrared spectroscopy, the modulus of elasticity in static bending, synergy interval partial least squares, successive projections algorithm, characteristic wavelengths

## Abstract

This article presents a non-destructive methodology to determine the modulus of elasticity (MOE) in static bending of wood through the use of near-infrared (NIR) spectroscopy. Wood specimens were obtained from *Quercus mongolica* growing in Northeast of China. The NIR spectra of specimens were acquired by using a one-chip NIR fiber optic spectrometer whose spectral range was 900~1900 nm. The raw spectra of specimens were pretreated by multiplication scatter correlation and Savitzky-Golay smoothing and differentiation filter. To reduce the dimensions of data and complexity of modeling, the synergy interval partial least squares and successive projections algorithm were applied to extract the characteristic wavelengths, which had closing relevance with the MOE of wood, and five characteristic wavelengths were selected from full 117 variables of a spectrum. Taking the characteristic wavelengths as input values, partial least square regression (PLSR) and the propagation neural network (BPNN) were implemented to establish calibration models. The predictive ability of the models was estimated by the coefficient of determination (*r_p_*) and the root mean square error of prediction (RMSEP) and in the prediction set. In comparison with the predicted results of the models, BPNN performed better results with the higher *r_p_* of 0.91 and lower RMSEP of 0.76. The results indicate that it is feasible to accurately determine the MOE of wood by using the NIR spectroscopy technique.

## 1. Introduction

*Quercus mongolica* is the main secondary forest species growing in Northeast China. *Quercus mongolica* is a frequently used structural material, also used for manufacturing of furniture, machinery, and sports appliances. The modulus of elasticity (MOE) in static bending is one of the most important mechanical properties of *Quercus mongolica*. It could be used in various ways or made into different kinds of products based on its MOE, so the detection of MOE can not only achieve its best use, but also ensure safe use in engineering. However, most of the traditional methods of testing mechanical properties of wood are destructive and time-consuming [1]. Although the results obtained are accurate, the test specimens after detection are usually no longer of use value, which causes great waste. Moreover, it is impossible to detect all the products, and the quality of the products cannot be guaranteed to meet the requirements. Therefore, the researchers propose to use non-destructive technology to determine the mechanical properties of wood.

Near-infrared (NIR) spectroscopy is a non-invasive analytical, high reliability, and pollution-free method to determine different properties of materials. NIR spectrum mainly reflects the second harmonic generation and co-frequency absorption of C-H, N-H, O-H and other hydrogen radical groups. The NIR absorption peaks of different groups have obvious differences. NIR spectra have abundant structural information and can be adapted to the determination of organic matters [2]. NIR spectroscopy has been studied by many scholars to determine the various properties of wood, such as moisture content [3], drying stress level [4], basic density [5]. In recent years, NIR spectroscopy technology has been widely studied on the determination of wood mechanical properties. For example, Schimleck et al. examined NIR spectroscopy for the estimation of MOE, and modulus of rupture (MOR) using clear wood samples obtained from several pine species; the results showed that NIR spectra collected from the radial and transverse faces provided similar calibration statistics [6]. Todorović, et al. predict the bending properties of thermally modified beech wood of both sapwood and red heartwood, the results of the spectra taken from sapwood were, in most models, better than the spectra of the red heartwood [7]. Acquah et al. incorporated tree breeding programs to further improve wood quality by estimating the mechanical properties and basic density of elite loblolly pine families by NIR spectroscopy [8].

The spectral analysis methods mainly focus on pretreatment methods, feature optimization, and prediction model establishment. Andrade et al. employed multiplication scatter correlation to pretreat the spectra before the prediction of MOE and the scattering light effect in the spectra of solid samples [9]. Liang et al. used backward interval partial least squares and genetic algorithm to extract the feature wavelengths from the original spectra to calibrate the model, which removed the noise and low information region of spectra and improved the prediction ability of the model [10]. When using NIR spectroscopy to analyze and predict the properties of wood, most of the models used were linear, such as principal component regression, multiple linear regression, and partial least squares regression (PLSR). Compared with the other linear regression methods, the predictive effects of PLSR was usually better [11]. Thus, PLS has become the main linear modeling method in the quantitative analysis of NIR spectroscopy. Besides, some of the non-linear modeling methods were applied in NIR spectroscopy, such as back propagation neural network [12], and support vector machines [13]. NIR spectroscopy combined with chemometrics analysis methods has the features of non-destruction and are efficient in predicting the mechanical properties of wood, which has a bright application prospect.

Therefore, the objective of this study was to investigate and build a new approach for quantitative analysis and determination of MOE in Static Bending of *Quercus mongolica* based on NIR spectroscopy combined with chemometrics analysis. The paper will focus on the following three aspects: (1) to reveal the relationship between NIR spectra and the MOE of the non-defect specimens of *Quercus mongolica*; (2) to explore an effective characteristic wavelengths extraction approach to select the closing relative information of NIR spectral data; and (3) to establish a model to predict the MOE in Static Bending of *Quercus mongolica* and evaluate the predictive ability of the model.

## 2. Materials and Methods

### 2.1. Specimen Preparation

The *Quercus mongolica* timber used in the experiment was collected from Chonghe forest farm, forestry bureau of Wuchang City, Heilongjiang province, China. The geographical coordinates were of 44°37′~44°47′ N, 27°35′~127°55′ E. The average elevation was 350 m. The climate of this area was temperate continental monsoon climate with annual temperature ranging from 35° to −34°, that led to an unfreezing period of 125 days. The annual precipitation and annual evaporation capacity were 750 mm, and 340 mm, respectively. In the *Quercus mongolica* section of the forest farm, 3 groups of trees were collected along the topography from high to low. Each group consisted of 4 trees, and 12 sample trees were obtained. In accordance with Chinese national standards, “General requirements for physical and mechanical tests of wood (GB1927~1943–2009)”, the timber was cut into small specimens with dimensions of 300 mm (L) × 20 mm (T) × 20 mm (R). 125 specimens without defects were chosen and numbered from 1 to 125. These specimens were then stored at constant temperature and in a constant humidity incubator; the moisture content of the incubator was adjusted to 12%. The NIR spectrum scanning and mechanical properties were tested in a laboratory. The temperature was 22 ± 1 °C and the average relative humidity was 50%.

### 2.2. NIR Spectra Measurements

**Figure 1:**
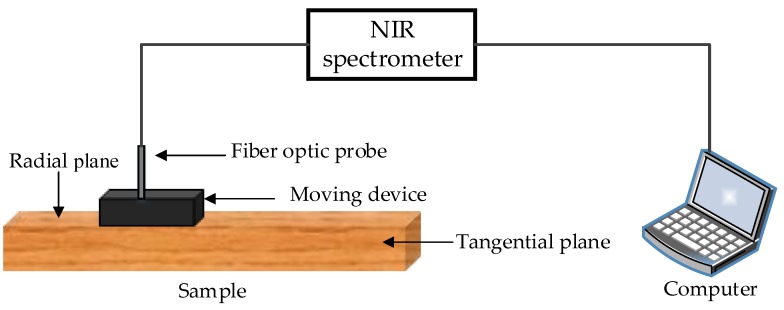
The diagram of spectra measurements.

In this work, the equipment used for specimen spectrum measurements was a One-chip NIR fiber optic spectrometer, which was researched and developed by Insion Co., GmbH, Heilbronn, Germany. The spectrometer utilized two bifurcated fiber optic probes to scan the diffuse reflectance spectrum in the surface of the specimen. The wavelength ranged from 900 to 1900 nm at a spectral resolution of 9 nm. Scholars and researchers found that spectroscopy in 1100 to 1700 nm contained important information that could be used to analyze and predict the properties of wood [7,14]. Before scanning the specimens, the spectrometer preheated for 10 min, and calibration was done with the commercial PTFE reference tile. The spectra of the specimens were then obtained. NIR spectra data were collected using SPEC view 7.1 software (Insion Co., GmbH, Heilbronn, Germany), and exported as Excel (Microsoft, Redmond, WA, USA). Each spectrum was acquired from an average of 30 scans. The process of spectra measurements is shown in Figure 1.

**Figure 2:**
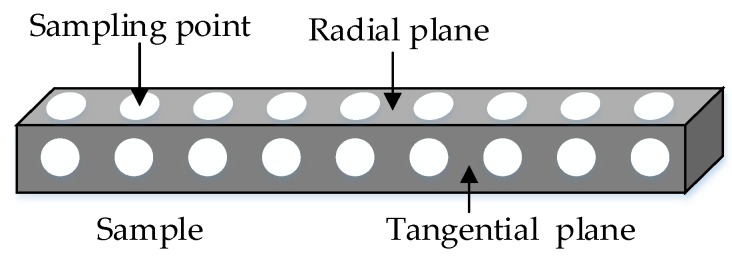
The spectral collection points of specimen.

Because of the difference of growth characteristics of timber, 9 spectra were collected uniformly in each radial plane and each tangential plane by moving the probe. The 36 spectra (18 from the radial plane and 18 from the tangential plane) were averaged to one single spectrum to represent the specimen they belonged to. Figure 2 shows the plane distribution and spectral collection points in the specimen.

### 2.3. Detemination of MOE in Static Bending

The experiment used the all-around mechanical testing machine of wood to determine the MOE in static bending of specimens, referring to steps and specifications in Chinese national standards, “Method for Determination of the Modulus of Elasticity in Static Bending of Wood (GB/T 1936.2-2009)”. All the samples were loaded in a tangential direction at a uniform rate (10 mm/min) until rupture (2 to 3 min per sample) to determine the MOE of the wood. The calculation of MOE was shown in Equation (1):

(1) $$MOE=\frac{23Pl^{3}}{108bh^{3}f}$$

where *P* was the difference of upper and lower limit load, *l* was the span of loading point, *b* was the breadth of specimen, *h* was the height of the specimen, and *f* was the deformation value in the middle of the specimen in the loading process.

### 2.4. Calibration Set and Predition Set Partitioning Using Improved Kennard-Stone Method

In order to guarantee the applicability and stability of the prediction model, the ratio of calibration set and prediction set was generally between 2:1 and 4:1. However, random set partitioning usually made the calibration set unrepresentative [15]. According to the spectral difference, the Kennard-Stone (K-S) method can put the biggest different sample into the calibration set and put the closing samples into the prediction set, ensuring the integrity and representativeness of the calibration set [16,17]. Since the traditional K-S algorithm calculated the Euclidean distance between any two samples in the sample pool in the high-dimensional space, the amount of calculation was very huge. Therefore, K-S algorithm was optimized by improving the distance formula of K-S in this work. The Euclidean distance used in the sample selection process of the K-S algorithm was replaced by the normalized Euclidean distance, improving computational efficiency. The formula calculating the spectral distance of samples in the improved K-S method is shown in Equation (2):

(2) $$d(p,q)=\frac{\sqrt{\sum_{j=1}^{m}{\left[x_{p}(j)-x_{q}(j)\right]}^{2}}}{\max\;\left\{d(p,q)\right\}};p,q\in [1,n]$$

where *x_p_*(*j*) and *x_q_*(*j*) were the absorbance of sample *p* and *q* at the *j* wavelength, respectively, *m* was the number of wavelengths in a spectrum, and *n* was the total number of samples. 

In this study, the ratio of the calibration set to the prediction set was chosen as 2:1.

### 2.5. Pretreatment of NIR Spectra

In the process of acquiring NIR spectrum data of the experimental samples, the noise, such as light scattering, high-frequency random noise, was inevitably caused by the spectrometer itself or the environment. The noise influenced the modeling effect and prediction accuracy. Therefore, the raw data needed to be pretreated before establishing the analysis model of the relationship between the NIR spectra and the mechanical properties of samples. The pretreatment methods studied in this work are multiplication scatter correlation (MSC), and Savitzky-Golay (SG) smoothing and differentiation filter.

MSC was used to compensate the dispersion effect of spectral data and reduce the occurrence of baseline drift. MSC was also used to correct the scattering of each spectrum and obtain an ideal spectrum [18]. SG smoothing, also called polynomial smoothing, was capable of eliminating the high-frequency noise, removing possible overlapping peaks, and correcting the spectra baseline [19]. The SG smoothing and differentiation filter did least-squares fitting of the data in the moving window through polynomials, so the smoothing effect varied by selection of window size. Besides, the spectral contour and absorption peak was more clear and obvious after pretreatment by using SG smoothing and differentiation filter, which did a fist derivative operation with the former spectra. Thus, the samples were pretreated by MSC combined with the SG convolution smoothing and differentiation filter.

### 2.6. Characteristic Spectrum Extraction

There was a lot of redundant information in the spectral data, which not only increased computational complexity, but also reduced the predicting accuracy of the model. Therefore, it was necessary to eliminate the uninformative wavelengths in the spectra that had unrelated MOE information. This process was named as characteristic spectrum extraction. The whole process included two steps: (1) optimal spectra intervals selection by synergy interval partial least squares (SiPLS); (2) characteristic wavelengths selection by successive projections algorithm (SPA).

#### 2.6.1. SiPLS

SiPLS was a further improvement on interval partial least squares (iPLS), proposed by Norgaard [20,21]. The basic principle of SiPLS was as follows: first, the full spectrum was divided into *N* smaller equidistant sub-intervals; second, the partial least squares (PLS) regression model was built with *m* sub-intervals; and lastly the combination of sub-interval spectrum, which had the lowest root mean square error of the cross-validation showed better performance [22].

#### 2.6.2. SPA

SPA used vector projection analysis to find the variable set containing the lowest redundancy information and minimized the collinearity between variables. SPA selected a few groups of strong representative variables from the original spectrum of the experimental samples, which contained the majority of the spectral information. It also eliminated redundant and repetitive information in the spectral variables to improve the prediction ability of the model [23,24]. The main procedures are summarized here: the maximum number of variables *N* to be selected were set. Starting from each variable, SPA yields *M* (total number of variables) sets of selection of *N* variables. The optimal initial variable and number of variable can be determined on the basis of the smallest root mean squared error of prediction in a validation set of multiple linear regression calibrations [25].

### 2.7. Model Evaluation Standard

The quality of the models was assessed using several common statistical measures [26]: the coefficient of determination (*r_c_* of calibration set, *r_p_* of prediction set), the standard error of cross-validation (SECV), the root mean square error of prediction (RMSEP), and the ratio of performance to deviation (RPD). The type of cross-validation was leave-one-out RPD, which was the quotient of standard deviation (SD) of the true value of prediction set and the RMSEP of prediction set. The selection of the final model was based on its predictability following a procedure which has already been successfully applied [27]. Generally, a good model should have higher values of *r_c_*, *r_p_*, and RPD, but a lower value of RMSEC and RMSEP.

## 3. Results and Discussion

### 3.1. Determination of the MOE and Dataset Partitioning

The MOE of the 125 specimens ranged from 10.43 GPa to 19.25 GPa. The samples were partitioned into a calibration set and a prediction set by the improved K-S method, and the K-S method was carried out by using MATLAB. The ratio of these two sets was 2:1, that was, 84 specimens were put into the calibration set and 41 into the prediction set. The partition of sets is shown in Table 1. Table 2 shows the statistical value of the MOE of the two sets.

### 3.2. Near-Infrared Spectra of Specimens and Spectral Pretreatment

**Figure 3:**
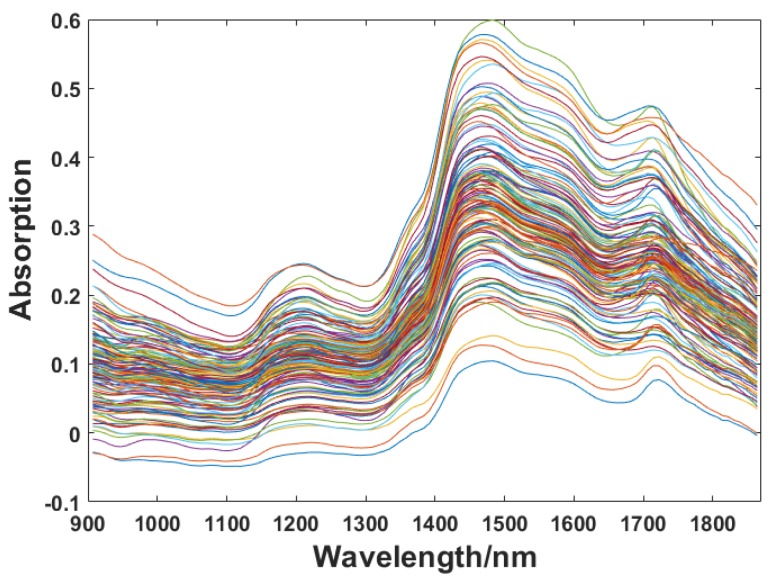
Raw spectra of specimens.

The NIR spectra of 125 specimens were collected with a wavelength ranging from 907 to 1864 nm. The number of sampling points of the wavelength variable was 117, and the raw spectra of all the specimens are as shown in Figure 3.

**Figure 4:**
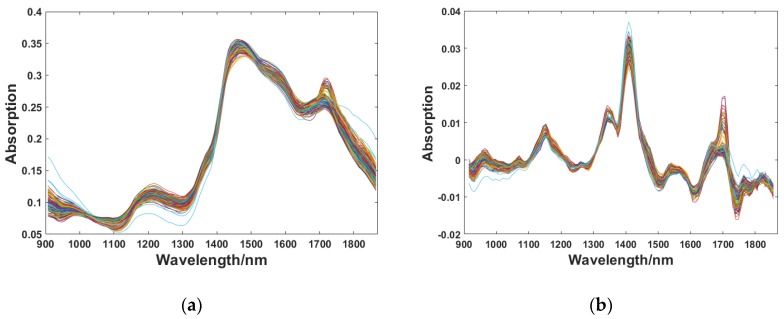
Pretreated spectra: (**a**) Pretreated by MSC; (**b**) Pretreated by MSC combined with SG convolution smoothing and differentiation filter.

The raw spectra were pretreated by MSC combined with the SG convolution smoothing and differentiation filter. The results of pretreatment are shown in Figure 4. It can be seen from Figure 4a that the influence factors of the scattered light were weakened after the raw spectra was corrected by MSC, and the spectral aggregation degree was stronger after the pretreatment. In addition, the trend of the change was more uniform. The spectral absorption peaks did not, however, become obvious, and the intensity of information was still low. Therefore, the SG convolution smoothing and differentiation filter were applied. It not only eliminated the effects of high-frequency noise, but also eliminated the baseline offset caused by environmental changes. The window size of the SG convolution smoothing algorithm is generally selected as 5, 7, 9, 11, and 13. On the basis of the experimental results, it was revealed that pretreatment effects were the best with window size of 11 and polynomial order of 3. The spectra pretreated by MSC combined with the SG convolution smoothing and differentiation filter is shown in Figure 4b. Figure 4b infers that the problems of raw spectra; including scattered light, baseline drift, and high-frequency noise had been solved after pretreatment, and the spectral information was enhanced.

### 3.3. Characteristic Spectrum Selection

#### 3.3.1. Optimal Spectra Intervals Selected by SiPLS

**Figure 5:**
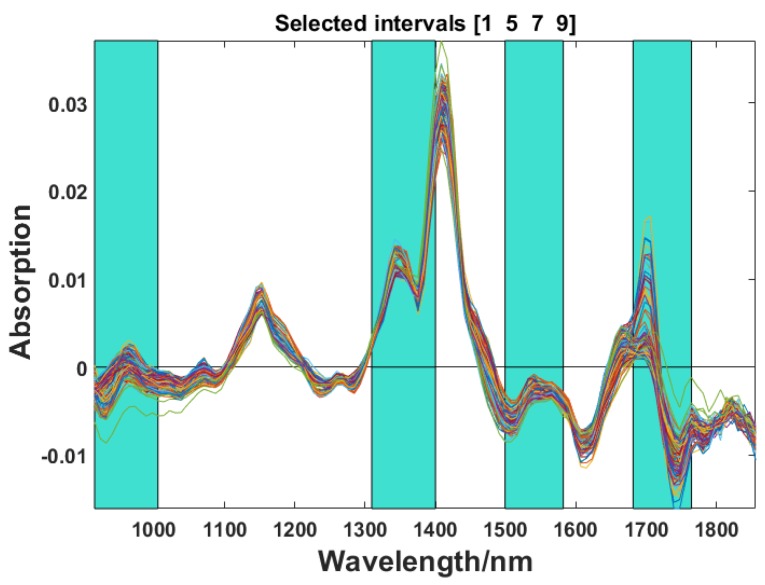
Optimal spectra intervals selected by SiPLS.

In this study, the full spectrum (907~1864 nm) of specimens was divided into 5, 6, …, 15 intervals combined with 2, 3 or 4 subintervals. The optimal combination of intervals and the number of PCs was optimized by full cross-validation and determined according to the lowest RMSECV. In this study, the optimal spectra intervals were a combination of four subintervals when the spectrum was divided into 10 subintervals. Table 3 shows the results of selected optimal spectral subintervals, and Figure 5 demonstrates the spectral regions corresponding to the optimal subintervals, i.e., 915.09~1005.2 nm, 1309.4~1400.1 nm, 1499.2~1581.9 nm, and 1681.3~1764.3 nm.

#### 3.3.2. Characteristic Wavelengths Selected by SPA

**Figure 6:**
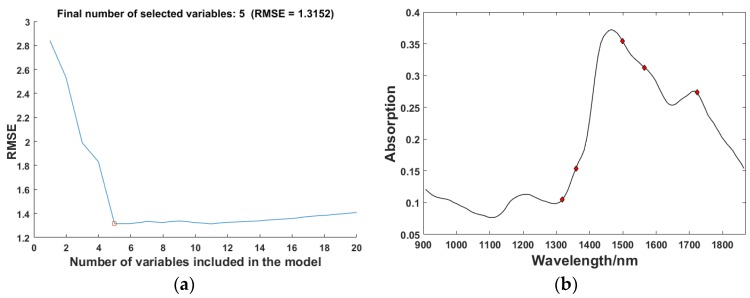
The results of characteristic wavelengths selection by SPA: (**a**) The variation of RMSE with SPA; (**b**) Final selected wavelengths.

After optimal spectral intervals selection by SiPLS, the number of wavelengths was reduced from 117 to 46. The 46 variables were numbered from 1 to 46. SPA was then implemented to select the characteristic wavelengths from the 46 variables. Figure 6a shows the relationship between the number of different variables and the root mean square error (RMSE). In the process of increasing the number of variables, the value of RMSE decreased significantly. When the number of variables was 5, the value of RMSE was the smallest at 1.3152. However, RMSE was rising when selected variables continue to increase. The numbers of variables selected were 14, 19, 25, 33, and 41. The distribution of the final selected wavelengths in one of the raw spectrums is shown in Figure 6b. The final characteristic wavelengths selected by SPA were 1317.6 nm, 1358.8 nm, 1499.2 nm, 1565.36 nm, and 1722.78 nm. According to the Ref [28], these five wavelengths were in the NIR spectra range, which would reveal the relationship between NIR spectroscopy and resin, cellulose, lignin, and hemicellulose. Since the contents of these organic components in the wood had a direct connection with the physical properties of the wood, the information in these characteristic wavelengths can be used to indirectly determine the MOE of the wood.

### 3.4. Analysis of the Predictive Models

**Figure 7:**
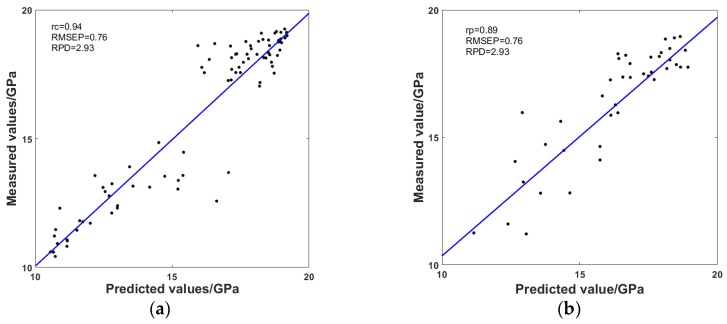
Relationships between the measured and predicted MOE of *Quercus mongolica* in the (**a**) calibration set and (**b**) prediction set.

The experiments used the characteristic wavelengths in the calibration set to establish a calibration model. Partial least square regression (PLSR) was the main linear modeling method in NIR quantitative analysis, so it was used to analyze the relationship between NIR spectrum and MOE of the specimen in this work. On the other hand, the propagation neural network (BPNN), which is a widely used nonlinear model, was also selected to predict the MOE of wood. Table 4 demonstrates the comparison of prediction effects of PLSR and BPNN. It can be concluded that BPNN obtained better predictive performance than PLSR, which was at a higher *r_p_* of 0.91 and lower RMSEP of 0.76. Furthermore, the RPD of BPNN was between 2.5 and 3.0, which suggested that the model met the needs of quantitative prediction [26]. The experimental results indicated that BPNN was capable of quantitative analysis and prediction of the MOE of *Quercus mongolica* specimens without defects. Figure 7 shows the relationship between measured and predicted MOE of *Quercus mongolica* specimens with BPNN as the prediction model. Figure 7 shows a clear separation of high and lower MOE values in the calibration set; this is because in order to ensure the integrity and representativeness of the calibration set, the improved K-S put the biggest different sample into the calibration set and the closing samples into the prediction set, and led the number of specimens in the high MOE value and low MOE value were more than the others.

## 4. Conclusions

This study revealed the relationship between the NIR spectrum and the MOE of *Quercus mongolica* specimens without defects. NIR spectroscopy was used to establish a quantitative analysis model for non-destructive testing of MOE of samples. Based on the experimental results, the following conclusions can be drawn:

(1) The improved K-S method can make the sample distribution uniform, and ensure that the calibration set is widely distributed
(2) By pretreating with MSC and the SG smoothing and differentiation filter, the overall variation trend of spectra was more consistent, and the contour of spectra was more clear. Moreover, the absorption peak is more obvious. When the window size of SG was of 11, the effect of pretreatment was the best
(3) SiPLS combined with SPA could extract characteristic wavelengths that had the closest relevance with the MOE of *Quercus mongolica*. It reduced the dimensions of the original data, decreasing the computation and reducing the complexity of the modelling process.
(4) Compared with the prediction results, BPNN was better capable of predicting the MOE of the specimens by using the characteristic wavelengths to establish the calibration model. The *r_p_*, RMSEP, and RPD of BPNN were 0.91, 0.76, and 2.93, respectively. The quantitative prediction effects of the model can meet the needs of actual industrial activities.

## Figures and Tables

**Table 1:** The results of sets partition by improved K-S method.

Sample Set	Serial Number of Samples						
Calibration set	2	3	4	6	9	11	12
	15	16	17	18	19	20	21
	22	23	25	26	27	28	29
	30	32	35	36	37	40	41
	43	44	45	47	48	49	50
	52	53	54	56	60	63	64
	66	67	69	72	74	75	76
	77	78	79	80	82	83	84
	85	86	87	88	92	93	94
	95	96	97	98	100	101	104
	105	106	107	108	111	112	113
	114	115	118	120	122	123	125
Prediction set	1	5	7	8	10	13	14
	24	31	33	34	38	39	42
	46	51	55	57	58	59	61
	62	65	68	70	71	73	81
	89	90	91	99	102	103	109
	110	116	117	119	121	124	

**Table 2:** Statistics of the Compressive Strength from the Calibration and Prediction Sets.

Samples	Maximum (GPa)	Minimum (GPa)	Mean (GPa)	Standard Deviation (GPa)
Calibration set (*n* = 84)	19.25	10.43	16.00	3.05
Prediction set (*n* = 41)	18.96	11.22	16.41	2.23

**Table 3:** The results of selected optimal spectral subintervals.

Number of Intervals	PCs	Selected Subintervals	RMSECV
5	8	[1 3 5]	1.439
6	7	[1 2 3 6]	1.431
7	6	[1 5 7 9]	1.354
8	8	[1 6 7]	1.388
9	8	[1 2 6 8]	1.355
10	6	[1 5 7 9]	1.354
11	7	[1 2 8 10]	1.374
12	8	[1 2 9 11]	1.360
13	6	[1 6 9 11]	1.387
14	7	[1 7 10 12]	1.388
15	7	[1 7 12 13]	1.389

**Table 4:** Comparison of the calibration model results with PLSR and BPNN.

Types of model	*r_c_*	RMSEC	SECV	*r_p_*	RMSEP	RPD
PLSR	0.90	1.35	1.34	0.84	1.08	2.06
BPNN	0.94	1.00	1.04	0.89	0.76	2.93
